# A capture method based on the VC1 domain reveals new binding properties of the human receptor for advanced glycation end products (RAGE)

**DOI:** 10.1016/j.redox.2016.12.017

**Published:** 2016-12-18

**Authors:** Genny Degani, Alessandra A. Altomare, Mara Colzani, Caterina Martino, Angelica Mazzolari, Guenter Fritz, Giulio Vistoli, Laura Popolo, Giancarlo Aldini

**Affiliations:** a University of Milan, Department of Biosciences, Via Celoria 26, 20133 Milano, Italy; b Department of Pharmaceutical Sciences, Via Mangiagalli 25, 20133 Milano, Italy; c University of Freiburg, Institute of Neuropathology Neurozentrum, Breisacher Straße 64, 79106 Freiburg, Germany

**Keywords:** AGEs and ALEs, RAGE, Pull-down assay, VC1 domain, Reactive carbonyl species, MDA

## Abstract

The Advanced Glycation and Lipoxidation End products (AGEs and ALEs) are a heterogeneous class of compounds derived from the non-enzymatic glycation or protein adduction by lipoxidation break-down products. The receptor for AGEs (RAGE) is involved in the progression of chronic diseases based on persistent inflammatory state and oxidative stress. RAGE is a pattern recognition receptor (PRR) and the inhibition of the interaction with its ligands or of the ligand accumulation have a potential therapeutic effect. The N-terminal domain of RAGE, the V domain, is the major site of AGEs binding and is stabilized by the adjacent C1 domain. In this study, we set up an affinity assay relying on the extremely specific biological interaction AGEs ligands have for the VC1 domain. A glycosylated form of VC1, produced in the yeast *Pichia pastoris,* was attached to magnetic beads and used as insoluble affinity matrix (VC1-resin). The VC1 interaction assay was employed to isolate specific VC1 binding partners from *in vitro* generated AGE-albumins and modifications were identified/localized by mass spectrometry analysis. Interestingly, this method also led to the isolation of ALEs produced by malondialdehyde treatment of albumins. Computational studies provided a rational-based interpretation of the contacts established by specific modified residues and amino acids of the V domain. The validation of VC1-resin in capturing AGE-albumins from complex biological mixtures such as plasma and milk, may lead to the identification of new RAGE ligands potentially involved in pro-inflammatory and pro-fibrotic responses, independently of their structures or physical properties, and without the use of any covalent derivatization process. In addition, the method can be applied to the identification of antagonists of RAGE-ligand interaction.

## Highlights

• A new VC1 interaction affinity assay was validated using model AGE-albumins.
• *In vitro* modifications of the interacting partners were identified/localized by MS.
• The VC1-pull down assays captures AGE-albumins in simulated complex mixtures
• ALEs produced by malondialdehyde treatment were mapped in VC1-interacting albumins.
• The molecular interactions of MDA-induced adduct-VC1 complexes were rationalized.

## Graphical abstract

**Figure:**
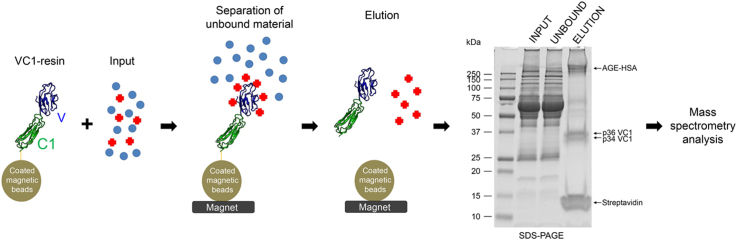

## 1 Introduction

Advanced Glycation and Lipoxidation End products (AGEs and ALEs) are now widely studied not only as biomarkers of oxidative stress but also because emerging data indicate their involvement as pathogenic factors and/or as damaging intermediates in several chronic diseases [1], [2], [3]. The identification, characterization and quantification of AGEs and ALEs in different physio-pathological conditions represent an emerging need, not only to find correlations with the onset and progression of diseases, but also to better understand the pathways dealing with their formation. From an analytical point of view, identification and characterization of AGEs and ALEs are very challenging and, despite the fact that several recent papers have increased our knowledge in this field, a detailed profile of these oxidation products is far from being reached for several reasons. AGEs and ALEs are chemically heterogeneous and this is due to the fact that, on one hand they arise from several reactive carbonyl species (RCS) chemically heterogeneous precursors which are formed by both lipid and sugar oxidation, and on the other hand RCS can react with different nucleophilic sites through multiple reaction mechanisms leading to a quite wide variety of reaction products including intra/intermolecular cross-links [4]. As an example, glyoxal, which is formed both from sugar and lipid oxidation, reacts with lysine, arginine and cysteine residues forming at least 11 reaction products including 3 cross-linked products.

Another aspect making the identification of protein adducts a quite complex matter is their presence in negligible amounts with respect to the native protein and their instability both from a metabolic and chemical point of view [5].

Because of the above mentioned aspects, most of the analytical methods so far proposed are based on a sample preparation procedure aimed at isolating/enriching AGEs and ALEs from native proteins followed by their identification and characterization by off target LC-MS approaches [6], [7]. Molecular recognition/enrichment based on immunoaffinity chromatography techniques is limited due to the chemical diversity of the analytes. Trapping AGEs/ALEs by chemical derivatization is also quite limited because most of the chemical moieties characterizing AGEs/ALEs are not suitable to be derivatized (not reactive), with the exception of the carbonyl group. Keto/aldehydic groups, which are characteristic of some protein adducts, are not contained in native proteins and they can be easily derivatized by using hydrazine and oxyl-amine containing agents [8]. Suitable enrichment columns targeting carbonyl functions have also been recently set-up and are found effective in separating carbonylated AGEs/ALEs from biological matrices [9], [10]. Carbonyl-based derivatization strategies have been used in some recent papers and they currently represent the state of the art procedure for identifying AGEs/ALEs in biological matrices [11], [12], [13].

**Fig. 1:**
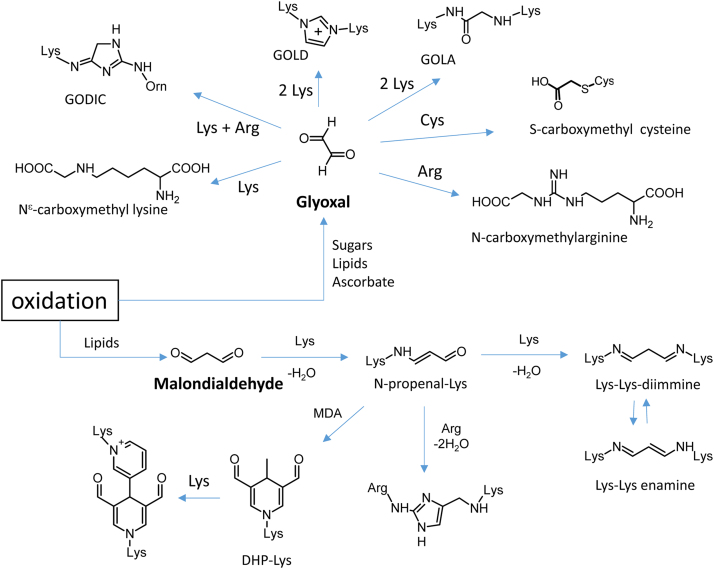
Major pathways for the formation of the glyoxal-derived AGEs and of MDA-derived ALEs.

However, we believe that such a strategy allows the identification of only a small fraction of AGEs/ALEs. This is due to the fact that, despite the carbonyl function being contained in most of the AGEs/ALEs precursors (RCS), it disappears once it covalently reacts with proteins, forming non-carbonylated reaction products. It should also be considered that protein carbonyls are still reactive and are quite unstable, as recently demonstrated by Liebler [5]. As an example, Fig. 1 shows the reaction pathways of malondialdehyde (MDA) and glyoxal with proteins, two RCSs whose formation has been demonstrated in several physio-pathological conditions [14]. MDA is in equilibrium with beta-hydroxy-acrolein which is an alfa-beta-unsaturated aldehyde forming a Michael adduct with nucleophilic sites such as lysine. N-propenal lysine can react with other nucleophilic sites such as lysine or arginine, forming cross-links that do not contain any carbonyl group or with another acrolein moiety forming di-hydropiperidine derivatives. Glyoxal is another example of aldehydic precursor which forms protein adducts most of them not containing derivatizable carbonyl moieties [4].

Based on such premises, we here propose a novel strategy of sample preparation that permits the modified proteins to be captured on the basis of their affinity for the VC1 domain of RAGE, one of the receptors that mediate their damaging biological effects. AGEs and ALEs can induce a damaging effect through different mechanisms such as loss of protein function or regulation, protein polymerization and aggregation, altered immune response and signaling due to activation of receptors such as RAGE. RAGE is a type I transmembrane glycoprotein and a member of the immunoglobulin (Ig) superfamily. It also shares structural similarities with a family of cell adhesion molecules (CAMs) [15]. RAGE-encoding gene (*AGER*) was originally isolated by a screening of a bovine lung cDNA library with an oligonucleotide probe based on the amino-terminal sequence of RAGE and the expression of the RAGE cDNA allowed transfected cells to bind ^125^I-AGE-albumin in a saturable and dose-dependent manner [16]. The ectodomain of RAGE is formed by three domains; V, C1 and C2. Although the recombinant V domain can be expressed in bacteria, purified and refolded *in vitro* and is the binding site for some ligands of RAGE, biochemical studies revealed that V and C1 form together a single integrated domain (VC1) that is the binding site for the majority of RAGE ligands [17], [18], [19]. We have shown in a previous report that VC1 can be produced as an autonomous domain in the methylotrophic yeast *Pichia pastoris* [20]. The glycosylation of VC1 and its secretion into the culture medium confers high solubility and stability to the protein, especially if compared to the protein expressed intracellularly in *E. coli* [20].

The idea of the herein proposed strategy has been to set-up an affinity chromatography based on a RAGE-VC1 stationary phase able to trap and then enrich/isolate modified proteins acting as RAGE ligands, independently of the modified moiety. The advantage of such an approach is that it is able to enrich functional AGEs and ALEs involved in pro-inflammatory and pro-fibrotic response through RAGE activation, independently of their structures and without the use of any covalent derivatization process.

## 2 Materials and methods

### 2.1 Strain and growth condition

*E. coli* DH5α strain was used for DNA manipulation. *P. pastoris* KM71 strain (Mut^S^, Arg^+^, His^-^) (Invitrogen) was used for the heterologous expression of VC1-His-Strep. *P. pastoris* cells were routinely propagated on plates of YPDA (1% yeast extract, 2% peptone, 2% glucose and 2% agar) at 30 °C. To induce the expression of recombinant proteins, His^+^ Mut^s^ cells were shifted from Glycerol-complex Medium (MGY) (1% yeast extract, 2% peptone, 1% glycerol, 1.34% YNB, 4×10^−5%^ biotin) to Methanol-Ethanol complex Medium (MEMY) (1% yeast extract, 2% peptone, 0.5% methanol, 0.7% Ethanol, 1.34% YNB, 4×10^−5%^ biotin). Cells were routinely grown in flasks at 30 °C under strong agitation and growth was monitored through the increase in optical density at 600 nm (OD_600_).

### 2.2 Construction of the expression plasmid

The recombinant plasmid for the expression of VC1-His-Strep in *P. pastoris* was obtained by cloning a *Xho*I-digested DNA fragment encoding the protein of interest into the corresponding sites of pHIL-S1 vector (Invitrogen). In this vector, the expression of the recombinant protein is under the control of the inducible *AOX1* promoter and the sequence encoding VC1 is in-frame with the secretion signal of *P. pastoris PHO1* encoding an extracellular acid phosphatase. The sequence of the primers used for the amplification of the DNA fragment were VCXhoIFOR (5′-agcatattcgactgactcgag*ctcaaaacatcacagcccg*−3′) and VC1-233His-StrepREV (5′-atcgtcgggctcactcgagCTA**accaccgaactgcgggtgacgcca**agcgctaccaccgctaccaccaccgctaccaccacc***gtgatggtgatggtgatg***ggcgct*cacaggctcccagacacg*−3′) where the *XhoI* site is underlined, the stop codon is in capitol letter, the sequence encoding the Strep tag (AWRHPQFGG) is in bold, the sequence encoding the 6x His tag is in italic and bold, the CDS of VC1 is in italic. The two tags are separated in the product by a linker of sequence GGGSGGGSGGSA. PCR was carried out using as a template pET-15b-VC1243, harbouring the cDNA encoding the precursor of hRAGE, and primers amplified a portion corresponding to the V and C1 domains (amino acid residues 23–243). PCR amplification was carried out using the Phusion® Hot Start High-Fidelity DNA Polymerase (New England Biolabs, NEB) and the DNA fragment was purified using Geneclean Turbo for PCR kit (QIAGEN). The PCR product and the pHIL-S1 vector were digested with *Xho*I (NEB), gel-purified and ligated (Quick Ligation Kit, NEB). After transformation of DH5α *E. coli* cells, clones with the properly oriented insert were identified by colony PCR using a universal 5′-AOX1 primer with VC1-233His-StrepREV and VCXhoIFOR with the 3′AOX1-primer. Recombinant plasmid DNA, named pHIL-S1-VC1-His-Strep, was purified. The in-frame fusion and the absence of random mutations in the insert were verified by DNA sequencing (BMR Genomics, Padova, Italy). HIL-S1-VC1-His-Strep plasmid, linearized with *Sal*I, was transformed into KM71 *P. pastoris* cells using the “EasyComp” chemical transformation method (Invitrogen) as previously described [20].

### 2.3 Protein expression and purification

To induce the expression of the recombinant protein, cells were grown overnight at 30 °C in MGY under strong agitation and at a ratio between volume of culture and capacity of the flask of 1:10. Then, an appropriate amount of cells was collected by centrifugation and cells were suspended in methanol-ethanol-containing medium (MEMY) at an initial OD_600_ of ~1. Growth was monitored by increase of OD_600_. Fresh methanol was added daily to 0.5% (v/v) final concentration. To monitor protein expression, supernatants obtained by centrifugation of 1 ml-aliquots of culture, withdrawn at intervals after induction, were flash-frozen and stored at −20 °C for subsequent SDS-PAGE analyses. Ten clones were screened to select the best producer.

In general, VC1-His-Strep was purified from the culture medium by a single-step protocol. The culture supernatant was collected after 72 h from induction by centrifugation of the culture at 4300×*g* for 15 min at 4 °C. The culture supernatant was filtered (Nitrocellulose 0.22 µm) and either frozen at −20 °C or immediately processed. If frozen, after thawing at 4 °C the culture supernatant was filtered again (Nitrocellulose 0.22 µm). Appropriate aliquots from stock solutions (3 M NaCl, 1 M HEPES and 1 M imidazole) were added to bring the culture supernatant to the final concentration of 300 mM NaCl, 20 mM HEPES and 20 mM imidazole whereas pH was adjusted to 7.4 with NaOH. The solution was applied on a HisPrep FF (16/10) Ni-Sepharose Fast flow column (GE Healthcare) connected to AKTAprime plus and equilibrated with Buffer A (20 mM HEPES, 300 mM NaCl, 20 mM imidazole pH 7.4). The column was washed with Buffer A until the A_280_ reached the base line and the protein was eluted with a linear gradient of Buffer B (20 mM HEPES, 300 mM NaCl, 1 M imidazole, pH 7.4). The fractions containing the purified VC1-His-Strep were collected, analyzed by SDS PAGE and then dialysed for 16 h at 4 °C against Buffer C (20 mM HEPES, 100 mM NaCl pH 7.1).

### 2.4 Preparation of sugar-derived AGE- albumins

Bovine serum albumin (Fraction V, sterile filtered, endotoxin-tested, Cat no. A9576) and recombinant human serum albumin (HSA) expressed in *P. pastoris* (Cat no. A7736) were obtained from Sigma. AGE-serum albumins were obtained as described by Valencia J.V. et al. [21]. The albumins were incubated with glucose for 3 months or with ribose for one week. After incubation with the sugar and removal of the unreacted sugar by diafiltration on Amicon Ultra- 0.5 ml (Cut off 10 K), the fluorescence emission spectra of 0.05–1 mg ml^−1^ AGE-BSA and control BSA was determined using a Fluorescence Spectrofotometer (Varian, Cary Eclipse) at 380 nm excitation wavelength with emission scan from 400 to 550 nm where a maximum of emission intensity at approximately 440 nm was registered for AGE-albumins.

### 2.5 In vitro generation of protein adducts by reactive carbonyl species

Recombinant HSA was dissolved in phosphate buffer (10 mM sodium dihydrophosphate NaH_2_PO_4_·H_2_O buffered with 200 mM disodium phosphate Na_2_HPO_4_·2H_2_O to reach pH 7.4) at a concentration equal to 100 μM (6.7 mg ml^−1^). Malondialdehyde (MDA) was purchased from Sigma (Cat no. 63287). HSA was incubated at 37 °C in a final volume of 1 ml containing 500 μl of HSA 100 μM and MDA (stock solution 1.26 M in phosphate buffer) at increasing concentrations (315 μM, 3.15 mM, 31.5 mM, 315 mM, and 630 mM) corresponding to protein: RCS molar ratios equal to 1:6.3, 1:63, 1:630, 1:6300, 1: 12,600, or an equal volume of phosphate buffer was added to HSA to be used as a control untreated sample. The reactions were stopped after 24, 48 or 72 h by diluting the excess of RCS by ultrafiltration, using Amicon YM10 filter units (Millipore, Milan, Italy) as previously described [22]. Samples were diluted to 0.125 mg ml^−1^ with Buffer C and used for the pull-down assay.

### 2.6 Preparation of the VC1-resin

Purified VC1-His-Strep was immobilized on streptavidin-coated magnetic beads by exploiting the affinity of the Strep tag for Streptavidin (Streptavidin Mag Sepharose™, GE Healthcare, cat. N. 28-9857-99). The binding capacity of the beads was 3 μg of VC1-His-Strep/10 μl of medium slurry. In general, 50 μg of purified VC1-His-Strep in 170 μl of buffer C were added to 5 μl of packed streptavidin coated-beads previously obtained by washing 50 μl of 10% slurry with Buffer C, and incubated for 1 h at 4 °C on a rotary mixer at low speed. The unbound material was carefully removed and the magnetic beads were washed with 500 μl of buffer C and immediately used or stored at 4 °C. For simplicity, the VC1 bound to the beads is referred to as the VC1-resin and the beads alone as the control resin.

### 2.7 VC1 pull-down assay

The VC1-resin was incubated for 1 h at 4 °C with 160 μl of AGE-BSA or AGE-HSA or untreated BSA or HSA at the concentration of 125 μg ml^−1^ in buffer C. The unbound material was carefully removed and the beads were washed twice with 500 μl of Buffer C. The elution was performed by boiling the beads for 5 min in 15 μl of SDS Sample Buffer 2X [0.125 M Tris-HCl, pH 6.8, 4% (w/v) SDS, 200 mM dithiothreitol, 20% (w/v) glycerol and 0.02% Bromophenol blue] and then with other 10 μl of the same buffer. The two eluates were pooled. The control resin was treated in the same way to detect not specific binding. The various fractions were analyzed by SDS-PAGE.

In a simulated complex biological mixture, we used milk treated as follows: 5% (w/v) of dried skimmed milk powder was dissolved in 20 mM HEPES, 100 mM NaCl, pH 7.1, incubated at 95 °C for 5 min and then filtered (0.22 µm) to remove aggregates. AGE-BSA was used to spike 160 μl of 5% milk at a final concentration of 125 μg ml^−1^. Serum was diluted to reach a protein concentration of about 0.8 mg ml^−1^. 160 μl of the diluted serum were spiked with AGE-BSA at the same concentration as described above. In the pull-down assay of albumins added to complex mixtures, Triton X-100 was added to Buffer C at a final concentration of 0.5%. The protein eluates were resolved by SDS-PAGE.

### 2.8 Electrophoretic procedures

The fractions obtained from pull-down experiments were analyzed by SDS PAGE. 20 μl of input, unbound and wash fractions were mixed with 5 μl of SDS Sample Buffer 5X [0.312 M Tris-HCl, pH 6.8, 10% (w/v) SDS, 500 mM dithiothreitol, 50% (w/v) glycerol and 0.05% Bromophenol blue] and heated for 5 min at 99 °C. Samples, including the eluate obtained as described in paragraph 2.7, were separated by SDS-PAGE on any Kd pre-cast polyacrylamide gels (TGX, Bio-Rad) and stained with Coomassie blue (Bio-Safe, Bio-Rad). Images were acquired using the calibrated densitometer GS-800 and analyzed by the software Quantity one (Bio-Rad).

### 2.9 AGE- and ALE-serum albumin digestion

Polypeptide bands corresponding to AGE-BSA, obtained by incubation with ribose or glucose, and ALE-HSA incubated with MDA and retained by VC1, were excised from gels. After a brief wash with 100 μl of 50 mM ammonium bicarbonate (Sigma Aldrich), gel pieces were incubated with 10 mM dithiothreitol at 56 °C for 30 min and then with 55 mM iodoacetamide (Sigma Aldrich) at room temperature for 45 min in the dark. In-gel digestion of AGE-BSA and ALE-HSA was performed by overnight-incubation at 37 °C with 1 µg of sequencing-grade trypsin (Roche) dissolved in 50 mM ammonium bicarbonate. ALE-HSA was also subjected to a second digestion by a sequencing-grade chymotrypsin (1 µg) for 7 h at 25 °C in the presence of calcium chloride (10 mM). Peptide mixtures were extracted by a 10 min-incubation with extraction solution (acetonitrile/trifluoroacetic acid/water; 30/3/67; v/v/v) and by an additional 10 min-incubation with 100% acetonitrile. The two extracts were combined and dried in a vacuum concentrator (Martin Christ.). Digested peptide mixtures were then dissolved in an appropriate volume of 0.1% formic acid for mass spectrometry (MS) analysis.

### 2.10 Mass spectrometry analyses

Five μl of solubilized peptides were loaded on a home-made nano-column (PicoFrit 75 µm ID, 12 cm-long, New Objective) packed with ReproSil-Pur 120 C18-AQ resin (1.9 µm, Dr. Maisch GmbH) by an Easy-nLC 1000 (Proxeon) controlled by the software Xcalibur (Thermo, version 3.1.66.10). Peptides were separated using a reverse phase two-step gradient (from 10% to 70% acetonitrile in 0.1% formic acid over 25 min) and on-line electrosprayed into a Q-Exactive mass spectrometer equipped with a nanospray ion source (both Thermo). The mass spectrometer operated in data dependent acquisition (DDA) mode, controlled by the software Xcalibur to acquire both full MS spectra in the *m*/*z* range 300–2000 (resolution 35,000 FWHM at *m*/*z* 200) and MS/MS spectra obtained by HCD of the 10 most abundant multi-charged ions. Automatic gain control (AGC) was set at 3×10^6^ for the acquisition of full MS spectra and at 1×10^5^ for MS/MS spectra. To avoid redundancy, precursor ions already selected for fragmentation were dynamically excluded for 15 s. Mono-isotopic precursor selection was enabled; singly and unassigned charged ions were excluded from fragmentation.

### 2.11 Identification and localization of protein adducts

The software Proteome Discoverer (version 1.3.0.339, Thermo Scientific, USA) was used to extract peaks from spectra and to match them to the BSA sequence (Uniprot P02769) or HSA sequence (Uniprot P02768). Trypsin or trypsin/chymotrypsin was selected as the cleaving proteases, allowing a maximum of 3 missed cleavages. Peptide and fragment ion tolerances were set to 5 ppm and 10 mmu, respectively. Cys carbamidomethylation was set as fix modification (+57.02147). Methionine oxidation was allowed as a variable modification in addition to several ribose-, glucose- or MDA-derived modifications listed in Table S1. As a quality filter, only peptide matches with “high” confidence (FDR≤0.01) were considered as genuine peptide identifications. For the localization of AGE/ALE-deriving modifications, the MS/MS spectra of modified peptides were manually inspected; for the confident mapping of the modification site, spectra were requested to match the expected ions (*b* and/or *y*) neighbouring the modified amino acid both at the N- and C-termini.

### 2.12 Computational studies

To better investigate the key factors governing the interaction between RAGE and its ligands, with particular attention to MDA-induced adducts on HSA, a docking study was undertaken. The recombinant HSA crystal structure was retrieved from PDB (Id: 4G03). Simulations focused on the monomer A that shows the higher percentage of residues falling in the allowed regions of the Ramachandran plot. In detail, the water molecules and the crystallization additives were removed while the hydrogen atoms were added and, to remain compatible with the physiological pH, arginine, lysine, glutamate and aspartate residues were considered ionized by default. The completed protein structure was then minimized keeping the backbone atoms fixed to preserve the resolved folding. The optimized HSA was used to generate the experimentally detected MDA-induced adducts, described in Dataset S1, by manually modifying the arginine and lysine residues as listed in Table 3 using the VEGA suite of programs [23]. The modified HSA structure was finally minimized by keeping fixed all atoms outside a 8 Å radius sphere around the adducted residues and the optimized protein structure undertook to following docking analyses.

Similarly, the resolved structure of the RAGE V-domain was retrieved from PDB (Id: 2mov). This structure was chosen since it is in complex with a hydroimidazolone adduct, thus allowing a precise definition of the binding site to be used in the following docking simulations [24]. Among the 20 reported NMR structures, that with the highest percentage of residues falling in the allowed regions of the Ramachandran plot was utilized. The selected frame was then minimized keeping the backbone atoms fixed and then the included ligand was deleted.

In order to simplify the calculations and to render them suitable also for a screening of huge libraries of protein adducts, docking simulations were focused on the protein environments of the adducted residues as obtained by generating pseudo-ligand structures composed of the adducted residue plus the neighbouring residues comprised within a 5 Å radius sphere. For each adducted residue, the calculations were repeated by considering the same surrounding sphere and the residue with or without the detected modification. The set of computational experiments is the subject of a manuscript submitted to *Data in Brief*. Docking simulations were performed by PLANTS focusing the search on a 12 Å radius sphere around the bound hydroimidazolone. For each pseudo-ligand, 10 poses were generated and ranked by ChemPLP score with a speed equal to 1 [25]. The best pose so obtained were then optimized and utilized for rescoring analyses. All mentioned minimizations were performed by using Namd with the CHARMM force field and the Gasteiger's atomic charges [26].

## 3 Results

### 3.1 Expression and secretion of VC1-His-Strep from Pichia pastoris

We expressed a recombinant form of VC1, named VC1-His-Strep, in *P. pastoris* that previously proved to be a suitable host for the expression and secretion of stable and soluble forms of VC1 domain from human RAGE [20]. The C-terminal end of VC1 was fused to a 6 x Histidine (His) tag and a Strep tag (AWRHPQFGG), separated by an internal spacer. The tandem fusion was designed to facilitate the purification procedure by use of affinity chromatography on Nickel-chelating resins whereas the Strep tag was chosen for performing the high-affinity binding of the purified domain to Streptavidin-coated beads.

**Fig. 2:**
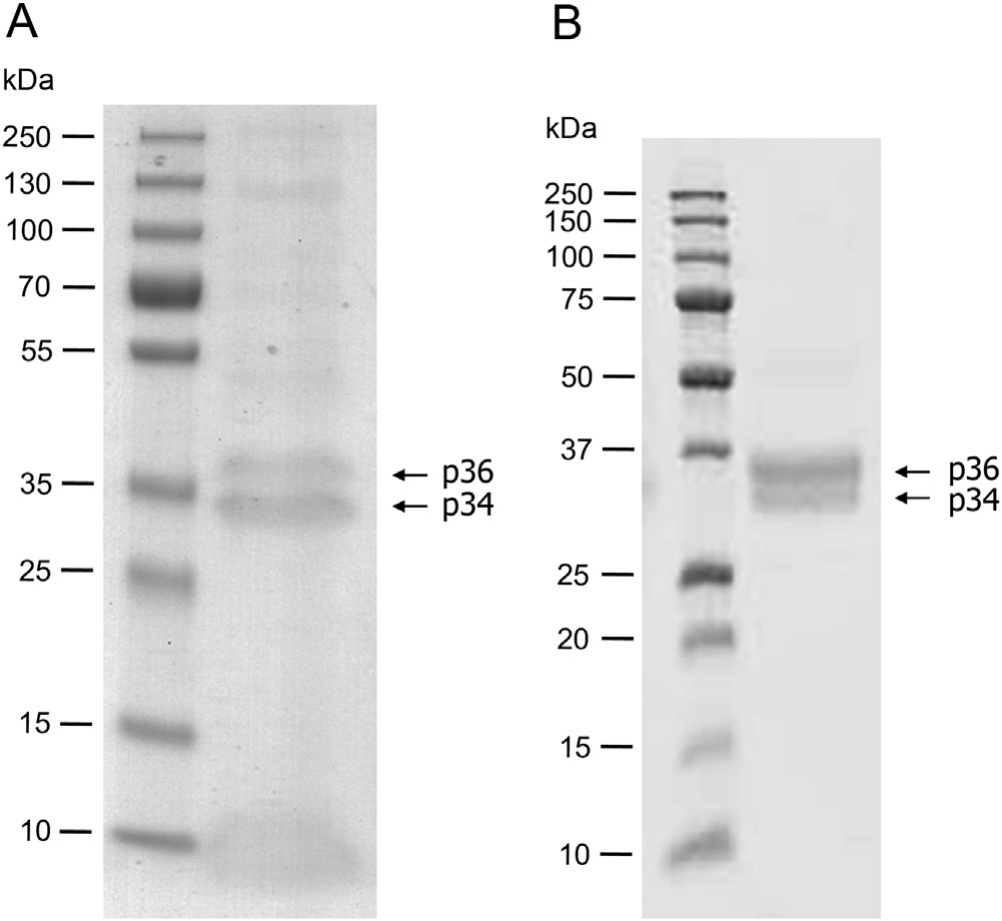
**One-step purification of VC1-His-Strep from the culture supernatant of*****P. pastoris*****recombinant cells**. (**A**) Culture supernatant was collected at 72 h after ethanol/methanol induction of KM71-VC1-His-Strep cells and an aliquot (30 μl) was directly analyzed by SDS-PAGE. (**B**) Secreted VC1-His-Strep (2 μg) was purified from the culture supernatant by Nikel-affinity chromatography. Proteins were separated by SDS-PAGE and stained by Coomassie blue. Arrows indicate the two glycoforms of VC1-His-Strep.

The adoption of a mixed ethanol/methanol induction protocol, described in Materials and methods, increased approximately 6 times the yield of secreted VC1-His-Strep compared to the previously protocol based on methanol alone [20]. At 72 h from the induction, VC1-His-Strep accumulated in the medium as two major polypeptide bands and the yield was about 45 mg liter^−1^ (Fig. 2**A**). One-step purification yielded homogeneous preparation of VC1-His-Strep with negligible loss of protein (<5%). As shown in Fig. 2**A** and **B**, VC1-His-Strep migrated as a doublet of two glycoforms, p36 and p34, consistently with the different degree of occupancy of two *N*-glycosylation sites, both occupied in p36 and occupied only at one site in p34, as described previously for untagged VC1 [20]. Finally, gel filtration studies yielded VC1-His-Strep that eluted in a single sharp peak corresponding to monomeric VC1 forms (data not shown). MS analysis of gel-purified VC1-His-Strep demonstrated the identity of the protein with the expected amino acid sequence, the proper removal of the signal peptide and the presence of the tags with coverage of 76.25%.

### 3.2 Functional characterization of immobilized VC1

Purified VC1-His-Strep was immobilized via the Strep tag on Streptavidin-coated magnetic beads and the derived VC1-resin was used as bait in pull-down assays.

**Fig. 3:**
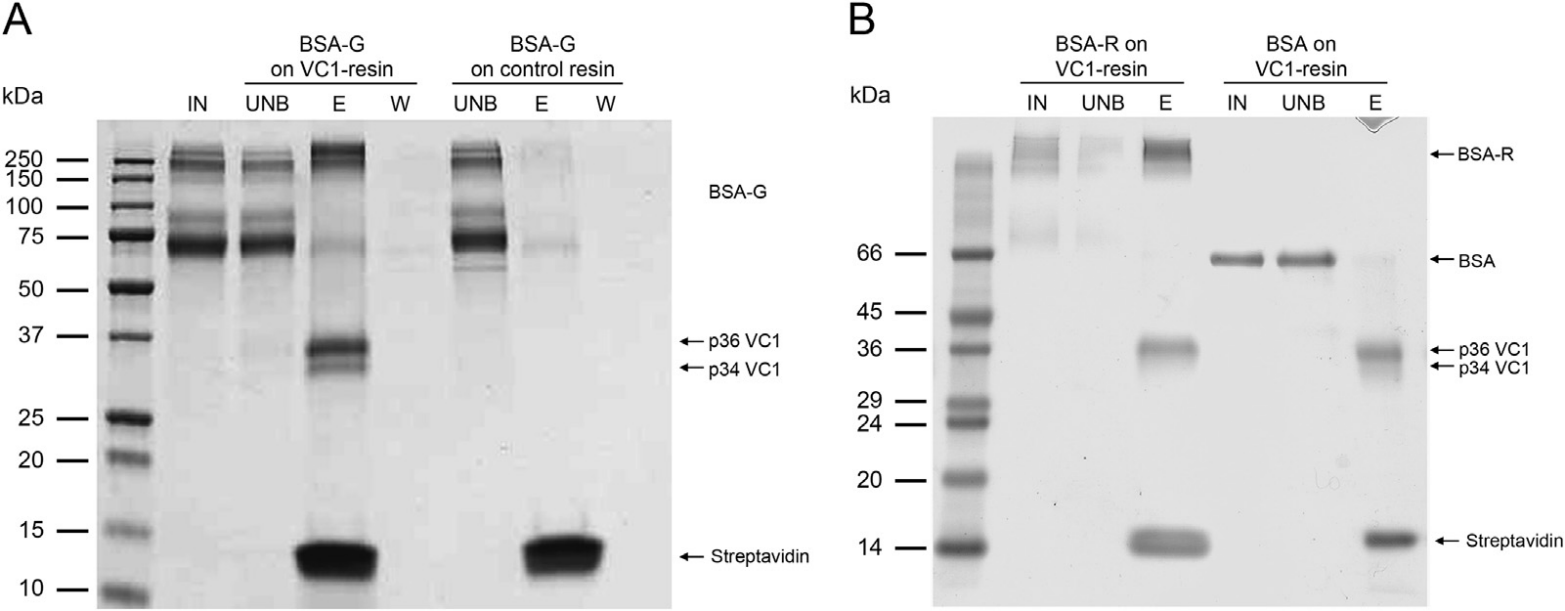
**Analysis of AGE-BSA binding by pull-down assay using the VC1-resin**. (**A**) Same amounts of AGE-BSA obtained by incubation with glucose (BSA-G) were used as input (IN). BSA-G was incubated with VC1 immobilized on the Streptavidin coated magnetic beads (VC1-resin) or with the Streptavidin-coated magnetic beads alone (control resin) as indicated. The unbound fraction (UNB), the second wash after binding (W) and the eluate (E) from each resin are shown. The E fraction is obtained by boiling the resin with SDS-sample buffer as indicated in Materials and methods and this step removes any associated molecule from the resin including the two forms of VC1 (p34 and p36), and Streptavidin (~14 kDa). Note that VC1 forms were not present in the eluate from the control resin because VC1 was not immobilized on these beads. (**B**) AGE-BSA obtained by incubation of BSA with ribose (BSA-R) or reagent-grade BSA were used as input (IN). Both samples were incubated with the VC1-resin and the unbound fraction (UNB) and eluate (E) were analyzed. Samples were analyzed under reducing conditions by SDS-PAGE and stained with Coomassie blue. High MW- species of BSA-G or BSA-R are enriched by the pull-down assay with the VC1-resin and no binding of unglycated BSA occurs on VC1-resin.

In order to test if VC1-resin captures AGEs, AGE-serum albumins obtained after prolonged *in vitro* incubation with reducing sugars, glucose or ribose, were used as preys in pull-down assays. Streptavidin-coated magnetic beads alone were used as a control (control resin). Optimal saline concentrations were determined by testing the effect of different concentrations of NaCl (from 20 to 100 mM) in the binding or/and washes. A concentration of 100 mM NaCl proved optimal for specific binding (data not shown). Binding of AGE-BSA was performed by incubating BSA obtained by reaction with glucose, called BSA-Glucose (BSA-G), with the VC1- or control resin. After removal of the unbound fraction and two washes, the complex formed by the bound protein-VC1 and Streptavidin was dissolved by boiling the resin in SDS-sample buffer. SDS-PAGE analysis of the eluates from the VC1-resin and the control resin showed the binding of BSA-G to the VC1-resin and no significant interaction with the control resin (Fig. 3**A**). As expected, streptavidin was present in the eluate from both the VC1-resin and the control resin whereas VC1 was only present in the eluate from the VC1-resin and not from the control resin (Fig. 3**A**). High molecular weight (HMW)-species of BSA-G (≥250 kDa), likely representing cross-linked and highly modified species, were preferentially retained by VC1.

**Fig. 4:**
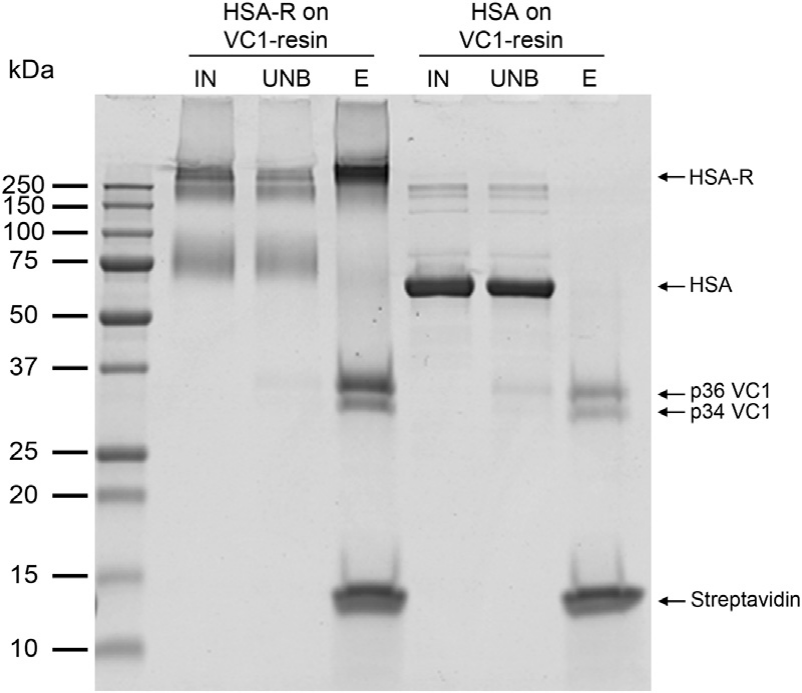
**Binding of AGE-HSA to the VC1-resin**. Recombinant HSA obtained by incubation in the presence (HSA-R) or absence of ribose (HSA), was used as input (IN). Both samples were used in pull-down assays with the VC1-resin and the unbound fraction (UNB) and eluate (E) were analyzed by SDS-PAGE, as described in Fig. 3. The gel shows that High MW species of HSA-R are preferentially retained by the VC1-resin whereas untreated HSA (66 kDa) does not bind VC1.

We also tested AGE-BSA obtained by incubation with ribose (BSA-R). Fig. 3**B** shows that VC1 enriches HMW-species present in the sample. Untreated BSA was not retained by the VC1-resin and migrated as a 66 kDa-polypeptide as expected for the unmodified albumin (Fig. 3**B**). No binding of BSA-R was detected with the control resin (data not shown). Additionally, recombinant human serum albumin treated with ribose (HSA-R) gave similar results (Fig. 4). In conclusion, VC1-resin specifically captures AGE-modified serum albumins.

### 3.3 Characterization by MS of AGE products retained by VC1

To determine the type of modifications present in the molecular species of AGE-BSA captured by the VC1 domain, a MS analysis of the in gel-digested HMW bands was performed (Fig. 3**A**, lane E from VC1-resin, bands ≥250 kDa). The localization of each modification was mapped in the amino acid sequence of BSA by using stringent criteria to avoid false positive hits. Thus, only high-confidence identification and mapping were taken into account. Table 1 shows that the most modified residues were Lys^256^ (Schiff base, deoxy-fructosyl- and carboxymethyl-) and Lys^437^ (carboxymethyl-, deoxyfructosyl-) indicating that these sites are the most susceptible to modifications following treatment of BSA with glucose.

Table 2 reports the modified peptides detected in HMW species of AGE-BSA obtained by incubation with ribose and retained by VC1 (Fig. 3**B**, *lane* E from VC1-resin, bands ≥250 kDa). Ribose treatment generated different adducts. The most frequently modified residues were: Lys^235^ (pyrraline and Schiff base), Arg^360^ (carboxymethyl-, Schiff base and +218 modification) and Lys^437^ (pyrraline, Schiff base, +218 modification and carboxyethyl-). Overall, Lys^437^ was particularly prone to modification by both sugars, indicating that is a highly reactive residue as previously reported [27]. In this study, both Lys^548^ and Arg^360^ were susceptible to modifications following treatment of BSA with either one of the two sugars although the type of modifications was different (Table 1, Table 2). Interestingly, Lys^548^ of BSA corresponds to Lys^549^ in HSA that was recently detected also *in vivo* as a target residue of an AGE modification in human serum albumin [28].

### 3.4 Capability of VC1 to capture modified albumins in complex biological matrices

The VC1-interaction experiments described above show the specific interaction of VC-resin with model AGE-proteins. To test the specificity of VC1 binding in complex mixtures, we spiked AGE-serum albumins in simulated biological samples such as milk and serum. The conditions of the pull-down experiments were the same except that 0.5% Triton X-100 was present in the binding and wash steps.

**Fig. 5:**
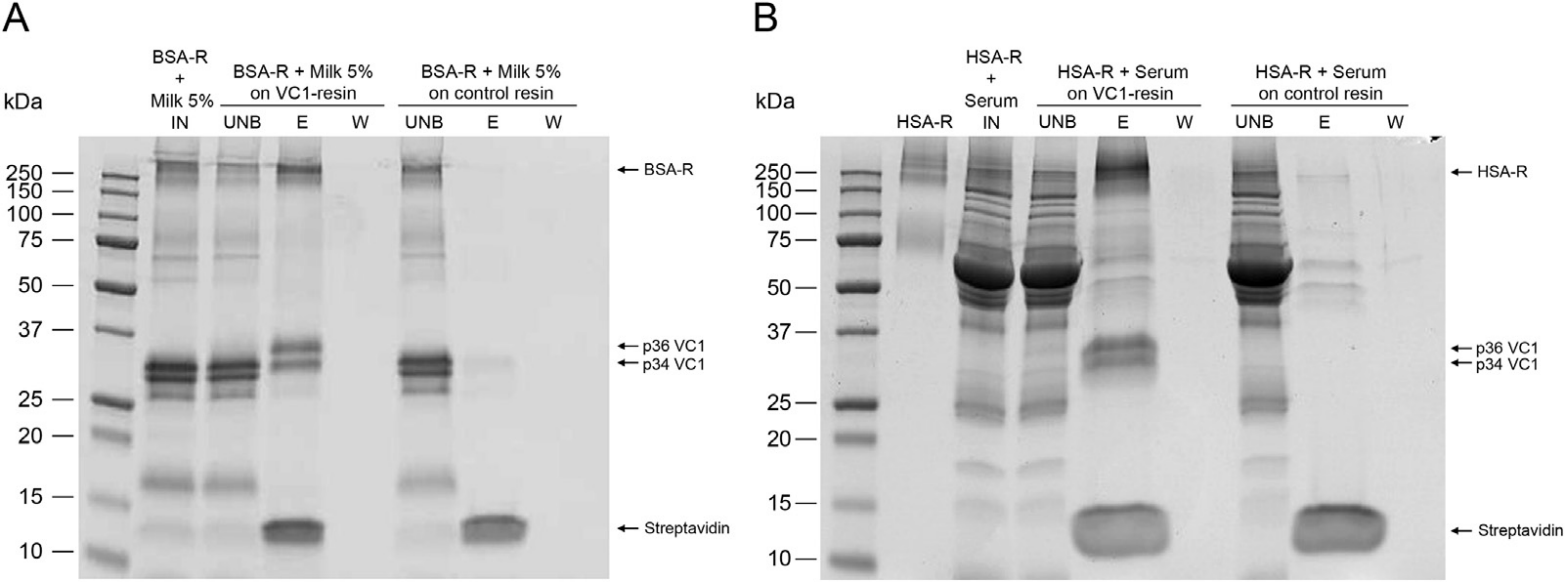
**AGE-serum albumin capture by VC1-resin in simulated complex matrices**. (**A**) BSA modified by incubation with ribose (BSA-R) was added to 5% milk (INPUT) and the simulated complex matrix was applied to the VC1-resin and to the control resin. (**B**) HSA modified by incubation with ribose (HSA-R, *first lane*) was added to human serum (INPUT, IN *second lane*) and the simulated complex matrix was applied to VC1-resin or to control resin. Unbound (UNB) and eluted (E) fractions were analyzed by SDS-PAGE, as described in Fig. 3. The gels show that HMW-species of AGE-serum albumins added to complex matrices are specifically retained by the VC1-resin.

We spiked a solution of 5% milk with AGE-BSA. Fig. 5**A** shows that VC1-resin specifically captures HMW-species of BSA-Ribose added to milk. A similar experiment was performed using human serum. Fig. 5**B** shows that AGE-HSA binds the VC1-resin in the presence of human serum and not the control resin. Thus, the VC1-resin is able to capture AGE-modified model proteins also in the presence of complex protein mixtures.

### 3.5 Binding of ALEs to VC1

**Fig. 6:**
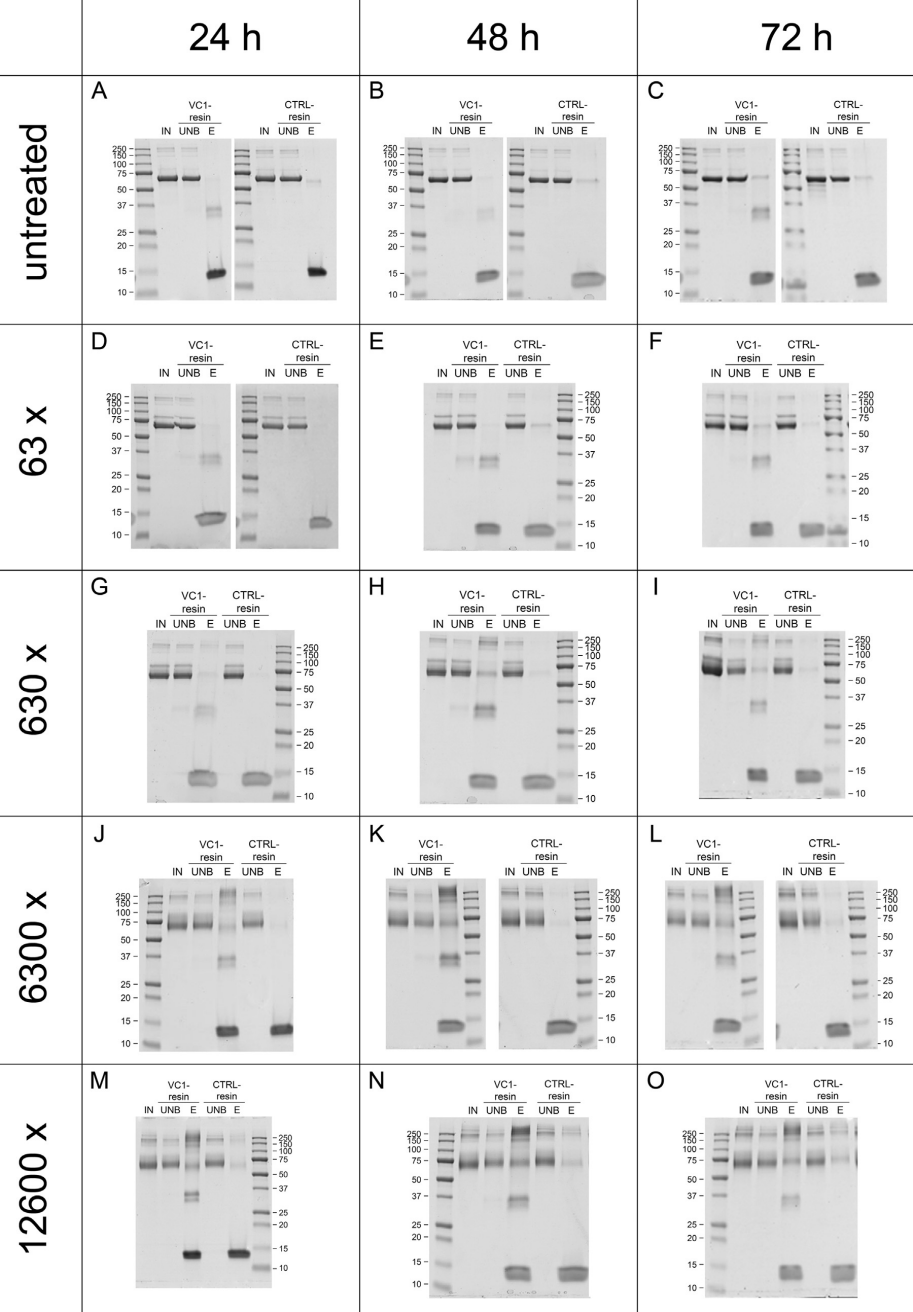
**Time course analysis of the dose-dependent modification of HSA by MDA**. Recombinant HSA was incubated with increasing concentrations of MDA as explained in Materials and methods. At 24, 48 and 72 h of incubation, aliquots were withdrawn and after removal of free MDA, pull-down assays with the VC1-resin or control (CTRL)-resin were performed as explained in Fig. 3. A specific binding of both the monomer of modified HSA and of the HMW-species to the VC1-resin was detected at increasing concentrations or time of incubation.

To further characterize the properties of VC1, we performed a time-course analysis of HSA incubated with different concentrations of MDA expressed in molar ration of MDA to HSA of 6.3, 63, 630, 6300 and 12,600. At 24, 48 and 72 h, untreated and treated HSA samples were assayed for binding to VC1-resins or to control resin. At 6.3 M ratio, binding of HSA to VC1-resin was negligible (data not shown). At increasing molar ratios and incubation time, higher amounts of HMW HSA species (≥250 kDa) and of a form of ~70 kDa were eluted from the VC1-resin but not from the control resin with a predominance of the HMW species *(*Fig. 6).

To characterize the nature of the modifications induced by MDA, the identified molecular species of HSA were subjected to in gel digestions and MS analysis (Dataset S1). Further computational studies based on these results are described in the next section.

Since MDA leads to the formation of ALEs, we can conclude that VC1 retains ALE-HSA, suggesting that RAGE is also a receptor for ALEs.

### 3.6 Computational results

Since interactions between RAGE and AGE have already been investigated at an atomic level (see for example the resolved and here utilized RAGE-AGE complex), we focused our attention only on the interactions between RAGE and MDA-induced adducts especially considering that this is the first study reporting RAGE-ALE interactions.

For each considered protein adduct and the corresponding unmodified residue, Table 3 compiles some representative docking scores plus the relative differences. In detail, Table 3 includes the ChemPLP score that was utilized by the docking program, the knowledge-based XScore plus two scores useful to reveal the specific contribution of ionic and non-ionic interactions, namely APBS which encodes for electrostatic contacts and LJ which encodes for van der Waals contacts as computed by the Lennard-Jones equation of the CHARMM force field. The results concerning Lys^65^, Lys^298^, Lys^499^ and Arg^281^ are not included in Table 3 since these residues are buried within protein pockets and thus the adducted residues were unable to conveniently approach the RAGE-VC1 surface. Gratifyingly, the compiled average scores and relative differences reveal that the adducted residues show better mean values in almost all considered scores compared to the unmodified ones, suggesting that the obtained docking results are able to rationalize the observed affinity of MDA-modified HSA for RAGE. When considering the specific scores, Table 3 reveals that the complexes for the adducted residues are substantially stabilized by ionic interactions, a result easily explainable considering that the simulated protein adducts involve the neutralization of a positively charged residue. Again, van der Waals contacts also play a clear stabilizing role which can be explained considering that the formed neutral adducts elicit non-ionic contacts with RAGE. When considering the score values for each simulated adduct, one may observe that Lys^75^ and Arg^361^ are the ones which show the most marked enhancement when comparing adducted and unmodified residues and focusing attention on both ionic APBS score and comprehensive scores (ChemPLP and XScore). Notably, the Arg^361^ adduct shows unfavourable van der Waals interactions, suggesting that here the ionic contacts elicited by the neutralization of an arginine residue are so favourable as to stabilize alone the complex.

**Fig. 7:**
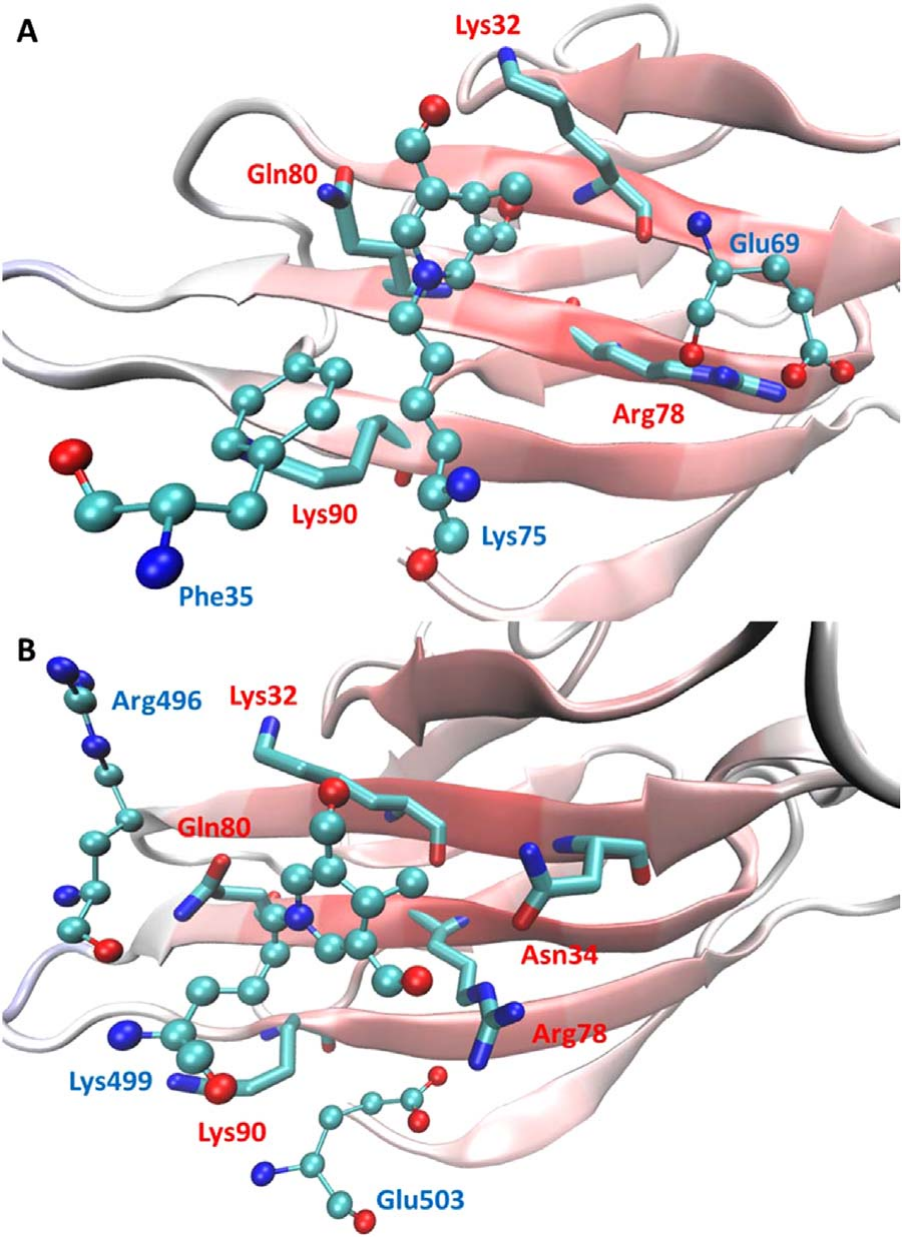
Comparison between the putative complexes in which the adducted residues affords the best (A) and the worst (B) stabilizing effect. In the figure, the protein cartoons refer to the V domain of hRAGE, the HSA residues are drawn in ball-and-stick and their numbering is blue colored, while the key V domain residues are drawn in licorice and their numbering is red colored. (For interpretation of the references to color in this figure legend, the reader is referred to the web version of this article.)

In order to offer a more detailed rationalization of the score profiles reported in Table 3, Fig. 7 compares the putative RAGE complexes for the two adducts affording the best and the worst stabilizing effect (namely on Lys^75^ and Lys^499^), respectively. The reported stabilizing contacts reveal that in both complexes the neutral adduct stabilizes similar H-bonds with Lys^32^ and Gln^80^. Again, both complexes are stabilized by ionic contacts involving Arg^78^ of RAGE. The key difference which can explain the worst scores as computed for Lys^499^ concerns the presence of Arg^496^ which is engaged in ionic repulsion with Lys^32^ of RAGE, while in the complex for Lys^75^ all key ionized residues of RAGE (Lys^32^, Lys^80^ and Arg^78^) are surrounded by HSA residues with which they can elicit beneficial contacts. Such a comparison seems to confirm the key role played in RAGE-protein interactions by ion pairs and suggests that the adducted residues which afford the best interactions with RAGE are those which are surrounded by electron rich regions. Clearly, the reported docking results also emphasize the stabilizing role exerted by H-bonds and other non-ionic contacts, a finding particularly evident here considering the neutral state of the simulated adducts. Nevertheless, the reported complex for Lys^499^ reveals that, even a single ionic repulsion is conceivably so penalizing as to prevent the stabilization of a suitable complex regardless of the number of elicited H-bonds.

## 4 Discussion

In this report, we described the use of VC1 domain of hRAGE to set up a pull-down assay and its successful validation in binding AGE-modified proteins. VC1 was designed to bind a streptavidin coated-resin through the C-terminal Strep tag in order to orient the N-terminal portion of the V domain to freely interact with the ligands. This tag permits the use of metallic ions to be avoided since it has been previously reported that AGE-BSA provides sites for metal-binding and therefore binding to Ni-resin, independently of VC1, can occur [29]. Moreover, this affinity assay exploits the higher stability and solubility of the glycosylated VC1 domain expressed in *P. pastoris* compared to the forms produced intracellularly in bacteria. The VC1 pull-down experiments described in this work show that physiologically relevant and well known RAGE ligands, such as AGE-modified serum albumins prepared by incubating the protein with two reducing sugars (ribose or glucose), are specifically captured by VC1 domain while the native forms do not. These results underline both the selectivity of the resin and the fact that we reached our first goal, which is the set-up of an affinity resin for RAGE binders. Moreover, the isolation of the protein adducts derived from native and not binder proteins, also permitted the protein adducts acting as RAGE interactors to be fully characterized by bottom-up experiments. MS studies revealed that Lys and Arg residues are the two nucleophilic sites of BSA undergoing the covalent modifications and that pyrraline, Schiff base, and negatively charged moieties, carboxyethyl- and carboxymethyl-lysine are the modifying residues. Although a deeper investigation of the binding mechanism is required, such information strengthens the importance of the electrostatic interaction between RAGE and its binders. In particular, taking into account that the binding surface of RAGE is positively charged, the identified modifications suggest that the structural features turning a non-protein binder (BSA) to a binder (AGE-BSA) involve the covalent modification of positively charged residues (Lys and Arg) to form neutral or negatively charged residues as in the case of the carboxylated residues. We then turned our interest to understanding whether also ALEs, the protein adducts formed by lipid peroxidation by-products, can be RAGE binders. MDA was selected as lipid-peroxidation by-product able to generate ALEs by reacting with HSA. Our results indicate that also ALE generated by MDA binds RAGE, providing the first evidence that ALE are direct interactors of VC1. The pull-down experiment was then used to isolate the HSA-MDA adducts as RAGE binders which were then analyzed by bottom-up experiments. A comprehensive and original computational study was performed and applied to the understanding of the experimental results in terms of molecular interactions with the V domain. Interestingly, the docking results, reported in Table 3, contribute to explain the interaction of specific MDA- induced HSA adducts with RAGE-V domain. One of the “best score” residues is Lys^75^ of HSA whose modified version is predicted to establish ionic contacts and Van der Waals interaction with Lys^32^, Gln^80^ and Arg^78^ of the V domain. On opposite a residue with a poor score does not show effective interactions and the presence of repulsion effects are visible in the computed complex (Fig. 7). Even though a precise validation of the predictive power of such results would require a clearly wider number of simulated proteins, the marked score differences between adducted and unmodified residues provide a preliminary validation of the here applied computational strategy. Even though the specific contribution of each adducted residue is unknown, docking results allow a clear differentiation between the simulated residues. These docking differences are reflected into meaningful differences when analysing the specific key contacts as seen in the putative complexes (Fig. 7).

Further studies should be carried out to evaluate the binding capacity of ALEs generated by the different RCS generated by lipid peroxidation and detected *in vivo* such as acrolein, HNE and GO as well as AGEs formed by reducing sugar oxidation products such as MGO.

In conclusion, we here described a double tagged recombinant VC1 domain of hRAGE that can be used to functionalize stationary phases for affinity chromatography techniques. The application of the VC1-interaction pull down assay here reported is only in its infancy since several applications can be considered. The first application consists of isolating and then characterizing RAGE binders from the sera of subjects affected by different pathological conditions and involving an inflammatory condition evoked by RAGE activation, such as diabetes and atherosclerosis. In view of this application, we tested the selectivity of the resin by spiking AGE-BSA in human sera and milk. The results obtained indicate that VC1-resin represents a selective affinity phase for isolating RAGE binders from complex matrices such as serum and that it would represent a suitable tool for a functional based enrichment of AGEs and ALEs. This novel enrichment strategy has several advantages in respect to that based on derivatization and based on biotin hydrazide or aldehyde/keto reactive probe [7], since it permits the protein adducts to bind on the basis of their biological interaction (RAGE ligand) and independently of their structure, as the derivatization process does. These studies require a successive validation of the function by testing the activation of RAGE in cell lines expressing RAGE and a reporter of the nuclear factor kappa-light-chain-enhancer of activated B cells (NF-κB) transcription activation.

Further applications of the resin will involve the set-up of high throughput methods for the measurement of the binding affinities and for the screening of compounds acting as RAGE antagonists, the latter representing a novel medicinal chemistry approach for the treatment of several diseases, including Alzheimer's disease, diabetic complications and chronic inflammation [30].

## Conflict of interest

GD, MC, LP, GA are co-authors of a patent application (WO2016IB52391 20160427) based on some of the results included in the present paper and entitled “Improved system for the expression of the receptor for the advanced glycation end products (AGEs) and the advanced lipid glycation end products (ALEs) and applications thereof”.

## Figures and Tables

**Table 1:** AGE-modified peptides identified by MS analysis in high MW-species of BSA-glucose captured by VC1-resin.

**Modification**	**ΔM**	**Peptide sequence**^a^	**Amino acid**^b^	**Charge**	**MH+**^c^	**ppm**
Schiff base	+132.04426	AEFVEVTK*LVTDLTK	K256	+3	1824.98307	−0.60
Deoxy-fructosyl-lysine	+162.05282	ATEEQLK*TVMENFVAFVDK	K568	+3	2361.15543	+0.97
		QNCDQFEK*LGEYGFQNALIVR	K420	+3	2691.27079	−0.45
		ADLAK*YICDNQDTISSK	K285	+3	2103.97409	−0.49
		AEFVEVTK*LVTDLTK	K256	+3	1854.99442	−0.17
		SLHTLFGDELCK*VASLR	K100	+4	2108.06777	−0.73
		K*VPQVSTPTLVEVSR	K437	+3	1801.98758	−1.70
		NYQEAK*DAFLGSFLYEYSR	K346	+3	2463.13309	−0.82
Tetra-hydro-pyridmidine	+144.04226	YTR*KVPQVSTPTLVEVSR	R436	+3	2204.19156	−0.21
Carboxymethyl	+58.00548	AFDEK*LFTFHADICTLPDTEK	K528	+3	2556.19626	−0.05
		AEFVEVTK*LVTDLTK	K256	+2	1750.94621	−0.67
		HLVDEPQNLIK*QNCDQFEK	K412	+3	2413.14438	−0.40
		LVNELTEFAK*TCVADESHAGCEK	K75	+3	2666.20639	−0.37
		LSQK*FPKAEFVEVTK	K245	+3	1808.97855	−0.45
		K*QTALVELLK	K548	+3	1200.72013	+0.21
		K*VPQVSTPTLVEVSR	K437	+3	1697.94120	−1.24
Carboxyethyl	+72.02113	R*HPEYAVSVLLR	R360	+3	1511.83170	−0.82

The high MW species of BSA-Glucose (≥250 kDa) shown in Fig. 3A were subjected to in-gel digestion.

a The K* or R* indicate the position of the modified residue.

b The numbering refers to the amino acid residue in the sequence of the precursor of BSA (containing the signal peptide).

c Experimentally determined monoisotopic mass.

**Table 2:** AGE-modified peptides identified by MS analysis in high MW-species BSA-ribose captured by VC1-resin.

**Modification**	**ΔM**	**Peptide sequence**^a^	**Amino acid**^b^	**Charge**	**MH+**^c^	**ppm**
Carboxymethyl	+58.00548	R*HPEYAVSVLLR	R360	+3	1497.81552	−1.18
Pyrraline-derived	+78.01056	K*VPQVSTPTLVEVSR	K437	+3	1717.94822	−0.10
		ALK*AWSVAR	K235	+3	1079.59861	−1.01
		FPK*AEFVEVTK	K248	+3	1372.71704	+1.64
Schiff base	+132.04426	K*VPQVSTPTLVEVSR	K437	+3	1771.98740	+3.00
		ALK*AWSVAR	K235	+3	1133.62992	−3.06
		R*HPEYAVSVLLR	R360	+3	1571.85331	−1.76
Unknown	+218.07903	K*VPQVSTPTLVEVSR	K437	+3	1858.01419	−1.43
		K*QTALVELLK	K548	+3	1360.79386	+0.32
		R*HPEYAVSVLLR	R360	+3	1657.89438	+2.14
Methyl imidazolone	+54.01056	YLYEIAR*R	R167	+3	1137.60450	−0.57
Carboxyethyl	+72.02113	K*VPQVSTPTLVEVSR	K437	+3	1711.95627	−1.57
		FK*DLGEEHFK	K36	+3	1321.64435	+1.51

The high MW species of BSA-ribose (≥250 kDa) shown in Fig. 3B were subjected to in-gel digestion.

a The K* or R* indicate the position of the modified residue.

b The numbering refers to the amino acid residue in the sequence of the precursor BSA that includes the signal peptide.

c Experimentally determined monoisotopic mass.

**Table 3:** Comparison of representative docking scores for the adducted and unmodified amino acid residues of HSA plus overall score means.

Name	Adducted^a^	LJ_CHARMM	APBS	CHEMPLP	XScore
		(kcal/mol)	(kJ/mol)	(kcal/mol)	(kcal/mol)
Lys75	DHPLys	−98.53	−64.30	−58.58	−9.03
Lys75	No	−42.41	−34.90	−34.06	−5.68
	**Difference**	**−56.13**	**−29.40**	**−24.52**	**−3.35**

Lys337	DHPLys	−29.22	−95.00	−58.48	−6.29
Lys337	No	−21.49	−58.98	−58.57	−5.11
	**Difference**	**−7.73**	**−36.02**	**+0.09**	**−1.18**

Lys499	DHPLys	−54.98	−33.30	−34.35	−5.93
Lys499	No	−27.52	−37.63	−39.64	−4.25
	**Difference**	**−27.46**	**+4.33**	**+5.29**	**−1.68**

Lys588	DHPLys	−22.42	−97.60	−47.77	−5.77
Lys588	No	−11.50	−80.89	−37.14	−4.90
	**Difference**	**−10.92**	**−16.71**	**−10.63**	**−0.87**

Lys598	DHPLys	−27.53	−72.20	−67.40	−5.56
Lys598	No	−33.47	−42.50	−63.55	−5.86
	**Difference**	**+5.94**	**−29.70**	**−3.85**	**+0.30**

Arg361	RP	−56.90	−52.30	−50.12	−8.62
Arg361	No	−60.09	−4.12	−25.43	−6.19
	**Difference**	**+3.18**	**−48.18**	**−24.69**	**−2.43**

Mean	DHPLYS + RP	−48.26	−69.12	−52.78	−6.70
Mean	NO	−32.74	−43.17	−43.07	−5.33
Mean	**Difference**	**−15.52**	**−25.95**	**−9.72**	**−1.37**

a Data are derived from Dataset S1 and refers to the modified residues in MDA-treated HSA.
