# Supplementary figures and images for: A capture method based on the VC1 domain reveals new binding properties of the human receptor for advanced glycation end products (RAGE)
Source: Redox Biol. 2016 Dec 18;11:275–85. doi: 10.1016/j.redox.2016.12.017 (PMC5198869; doi:10.1016/j.redox.2016.12.017)

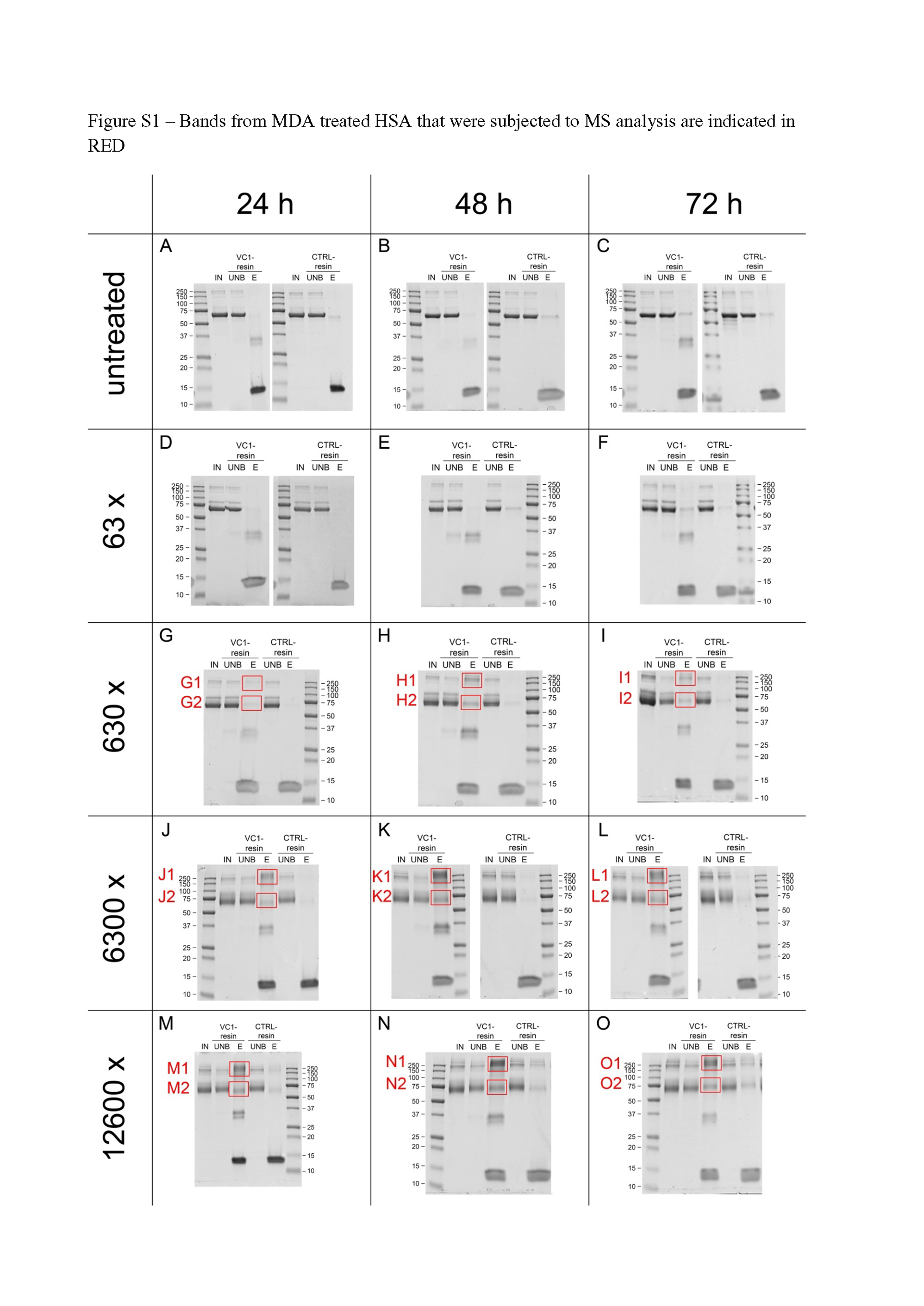

Supplement: Supplementary file 2 — Supplementary Material [file mmc2.zip › mmc2.tif]
