# Supplementary material for: A capture method based on the VC1 domain reveals new binding properties of the human receptor for advanced glycation end products (RAGE)
Source: Redox Biol. 2016 Dec 18;11:275–85. doi: 10.1016/j.redox.2016.12.017 (PMC5198869; doi:10.1016/j.redox.2016.12.017)
Supplement: Supplementary file 3 — Supplementary Material [file mmc3.doc]

**Supplementary Table S1**

List of the variable modifications searched in the peptides

| **AGE-BSA (glucose)** | | |
| --- | --- | --- |
| **Modification** | **Amino acid** | **ΔM** |
| Schiff base | K, R | + 132.04426 Da |
| Pyrraline-derived | K, R | + 78.01056 Da |
| Unknown | K, R | + 218.07903 Da |
| Deoxy-fructosyl-lysine | K, R | + 162.05282 Da |
| Pyrraline | K | + 108.17230 Da |
| Tetra-hydro-pyridmidine | R | + 144.04226 Da |
| Carboxymethyl | K, R | + 58.00548 Da |
| Imidazolone | R | + 39.99492 Da |
| Carboxyethyl | K, R | + 72.02113 Da |
| Methylimidazolone | R | + 54.01056 Da |
| **AGE-BSA (ribose)** | | |
| **Modification** | **Amino acid** | **ΔM** |
| Schiff base | K, R | + 132.04426 Da |
| Pyrraline-derived | K, R | + 78.01056 Da |
| Carboxymethyl | K, R | + 58.00548 Da |
| Unknown | K, R | + 218.07903 Da |
| Argpyrimidine | R | + 80.02622 Da |
| Tetra-hydro-pyridmidine | R | + 144.04226 Da |
| Methylimidazolone | R | + 54.01056 Da |
| Imidazolone | R | + 39.99492 Da |
| Carboxyethyl | K, R | + 72.02113 Da |
| **ALE-HSA (MDA)** | | |
| **Modification** | **Amino acid** | **ΔM** |
| N-propenallysine (NPK) | K | + 54.01056 Da |
| Dihydropyridine-lysine (DHPK) | K | + 134.03678 Da |
| Malondialdehydeargpyrimidine (RP) | R | + 36.0000 Da |
